# Selection and Characterization of a DNA Aptamer Recognizing High Mobility Group Box 1 Protein (HMGB1) and Enhancing Its Pro-inflammatory Activity

**DOI:** 10.5812/ijpr-147246

**Published:** 2024-09-11

**Authors:** Hanchao Li, Wengang Sun, Yanhua Huang, Qian Li, Hong Tian, Zhiming Hao, Yongwei Huo

**Affiliations:** 1 Department of Rheumatology, The First Affiliated Hospital of Xi’an Jiaotong University, Xi’an, Shaanxi Province, The People’s Republic of China; 2 Department of Rheumatology, Weihai Central Hospital, Weihai, Shandong Province, The People’s Republic of China; 3 Department of Gastroenterology, Affiliated Haimen Hospital of Xinglin College, Nantong University, Nantong, Jiangsu Province, The People’s Republic of China; 4 Department of Anatomy, Health Science Center, Xi’an Jiaotong University, Xi’an, Shaanxi Province, The People’s Republic of China

**Keywords:** Toll-Like Receptor 9 (TLR9), High Mobility Group Box 1 (HMGB1), Aptamer, Systematic Evolution of Ligands by Exponential Enrichment (SELEX), Inflammation

## Abstract

**Background:**

High mobility group box 1 (HMGB1) plays an essential role in various pathological conditions, including inflammation, fibrosis, autoimmune diseases, and carcinogenesis. The quantification of HMGB1 in body fluids holds promise for clinical applications.

**Objectives:**

This study aimed to isolate high-affinity single-stranded DNA (ssDNA) aptamers that target HMGB1.

**Methods:**

In this study, ssDNA aptamers were selected using Systematic Evolution of Ligands by Exponential Enrichment (SELEX). The affinity and specificity of the aptamers were evaluated through South-Western blot analysis, enzyme-linked aptamer sorbent assay (ELASA), and aptamer-based histochemistry staining. The impact of the aptamers on the biological activity of HMGB1 was tested in the human acute monocytic leukemia cell line, THP-1.

**Results:**

An aptamer (H-ap25, dissociation constant = 8.20 ± 0.53 nmol/L) with high affinity for the HMGB1 B box was generated. Further experiments verified that H-ap25 can be used to detect HMGB1 in South-Western blot analysis, ELASA, and aptamer-based histochemistry staining. Moreover, H-ap25 significantly augmented HMGB1-induced expression of tumor necrosis factor-α (TNF-α), interleukin (IL)-1β, IL-6, Toll-like receptor 9 (TLR9), and activation of NF-κB in THP-1 cells.

**Conclusions:**

Our results demonstrated that H-ap25 can be used both as an enhancer of HMGB1 and as a probe in research.

## 1. Background

High-mobility group box 1 protein (HMGB1) was first identified in 1973 as a highly conserved non-histone chromosomal protein and recognized as a structural protein extensively involved in DNA structuration, replication, transcription, and repair (1). In 1999, Wang et al. found that murine and human macrophages/monocytes released large amounts of HMGB1 upon exposure to bacterial endotoxin and subsequently verified that extracellular HMGB1 was crucially implicated in delayed endotoxin lethality as a pivotal mediator (2). Since then, the functions of extracellular HMGB1 as a cytokine-like protein have been widely studied. HMGB1 can be actively secreted by activated inflammatory cells via an unconventional pathway or passively released from damaged or necrotic cells (3). Extracellular HMGB1 acts as a damage-associated molecular pattern (DAMP) and plays important roles in the pathogenesis of various diseases, such as sepsis, arthritis, inflammatory bowel diseases, fibrotic diseases, diabetes, and malignancies, by activating the innate and adaptive immune response (2-11). Therefore, extracellular HMGB1 can be used as a novel diagnostic and prognostic marker as well as a therapeutic target for inflammatory diseases (3, 5, 10, 12, 13).

HMGB1 is ubiquitously expressed in almost all eukaryotic cells, and its amino acid sequence is highly conserved across species (over 98% identical between rodents and humans) (1). It consists of 215 amino acid residues with a calculated molecular weight of 24.9 kDa and structurally contains three distinct domains termed the A box (aa 1-85), B box (aa 88-162), and an acidic amino acid-rich (aspartic acid and glutamine) C-terminal tail (aa 186-215) (14, 15). Structure-function analysis has revealed that the cytokine-like activities of HMGB1 principally reside within the B box, while its A box domain acts as a natural competitive antagonist of the B box (5, 14, 15). The C-terminal acidic tail regulates the specific binding of HMGB1 to DNA, enhances the anti-inflammatory activity of the A box, and has an antibacterial function (16, 17).

Aptamers are short, single-stranded nucleic acid (DNA or RNA) sequences isolated from DNA/RNA libraries through a technology termed Systematic Evolution of Ligands by Exponential Enrichment (SELEX), which was devised in 1990 by three independent laboratories (18-20). Similar to antibodies, aptamers have specific higher-order structures and can bind to their targets with high affinities. However, aptamers offer some advantages over antibodies as they exhibit high thermal stability, rapid tissue penetration, and non-immunogenicity. Moreover, aptamers can be conveniently and inexpensively synthesized and modified. To date, numerous specific aptamers have been selected against a wide range of targets, including organic molecules, proteins, and even cells. Many aptamers have been used in biomedical research as molecular probes, inhibitors, or agonists. Recently, progress in nanotechnology has facilitated the applications of aptamers in precision medicine, and some nanoparticle-conjugated aptamers have been applied in cancer imaging and therapy (21-26). For example, the anti-VEGF165 RNA aptamer (Pegaptanib) has been successfully used in clinical practice for the treatment of ocular vascular diseases (25).

In this study, a single-stranded DNA (ssDNA) aptamer targeting the HMGB1 B box (H-ap25) was generated using SELEX. Further experiments showed that H-ap25 can be used as a probe to detect HMGB1 in South-Western blot analysis, enzyme-linked aptamer sorbent assay (ELASA), and aptamer-based histochemistry. Moreover, in vitro experiments demonstrated that H-ap25 drastically enhanced the pro-inflammatory activity of HMGB1. These data suggest that H-ap25 might be developed as both an enhancer and a probe for HMGB1 in research.

## 2. Objectives

This study aimed to select high-affinity ssDNA aptamers against HMGB1 and explore their application value.

## 3. Methods

### 3.1. Expression and Purification of Recombinant Human HMGB1(rhHMGB1), rhHMGB1 A Box and rhHMGB1 B Box

The coding regions of full-length human HMGB1, HMGB1 A box, and HMGB1 B box were obtained by PCR amplification using a plasmid carrying human HMGB1 cDNA (Sino Biological Inc, Beijing, China) with the primers listed in Table 1. The PCR products were cloned into pET28-a(+) to generate pET28a-HMGB1, pET28a-HMGB1 A box, and pET28a-HMGB1 B box. The recombinant expression plasmids were then transformed into *E. coli* BL21 (DE3) for expression. The recombinant proteins were purified by Ni-NTA affinity chromatography under native conditions. Bacterial endotoxin was removed using Triton X-114, as previously described (27).

**Table 1.** Primers for hHMGB1, HMGB1 A Box and B Box

Names	Sequences
**hHMGB1**	5′-CCCGAATTCATGGGCAAAGGAGATCCTAA-3′
	5′-CCCCTCGAGTTATTCATCATCATCATCTTC-3′
**hHMGB1 A-box **	5′-CCCGAATTCCCGAGAGGCAAAATGTCATC-3′
	5′-CCCCTCGAGGATATAGGTTTTCATTTCTC-3′
**hHMGB1 B-box**	5′-CCCGAATTCCCCAAGAGGCCTCCTTCGGC-3′
	5′-CCCCTCGAGTCGATATGCAGCAATATCCT-3′

### 3.2. SELEX Procedure, Cloning, DNA Sequencing and Secondary Structure Prediction

Recombinant human HMGB1 A box, rhHMGB1 B box, rhHMGB2 (Ag7989, purchased from Proteintech, Wuhan, China), and rhHMGB3 (Ag26361, Proteintech, Wuhan, China) were used to coat the microplate as the target proteins (2 pmol/well). The ssDNA library with a central region containing 40-nucleotide (nt) random sequences flanked by PCR primers on either side (Table 2) was synthesized as described in our previous study (28). In the 15-round SELEX, rhHMGB1 B box was used as the target protein, while rhHMGB1 A box, HMGB2, and HMGB3 were used as negative selection proteins. The final pool of dsDNA was cloned into the pGEM-T easy vector for isolating single colonies and sequence analysis. Asymmetric PCR using a biotin-labeled forward primer was performed to produce biotin-labeled aptamer candidates for further binding assays. RNA Structure v3.5 (Mathews Lab, Rochester, NY, USA) was used to predict the secondary structure of the ssDNA aptamer.

**Table 2.** The ssDNA Library and Primers for SELEX

Names	Sequences
**ssDNA library**	5′-GGACAAGAATCACCGCTC-N40-CGTACAGGAGGCATACAG-3′
**Forward primer**	5′-GGACAAGAATCACCGCTC-3′
**Reverse primer**	5′-CTGTATGCCTCCTGTACG-3′

### 3.3. Aptamer Binding Assay

Ninety-six-well polystyrene microplates were coated with 2 pmol rhHMGB1 A box, rhHMGB1 B box, or rhHMGB1 (diluted in 50 mM carbonate buffer, pH 9.6) and incubated at 4℃ overnight. The plates were then blocked with 3% BSA (blocking buffer) at 37℃ for 2 hours. H-ap25-biotin or library ssDNA-biotin (both at 5 nmol/L) in SHMCK buffer (20 mM Hepes, 120 mM NaCl, 5 mM KCl, 1 mM MgCl_2_, and 1 mM CaCl_2_, pH 7.4) was added to the microplates. After a one-hour incubation at 37℃ and four washes with phosphate-buffered saline containing 0.05% Tween-20 (PBST), streptavidin-HRP (1:5000 dilution, Bioss, Beijing, China, cat. Bs-0437P-HRP) was added to the microplates. The plates were incubated at 37℃ for 1 hour, followed by another four PBST washes. Finally, chromogenic solution (TMB/H_2_O_2_) was added, and absorbance at 450 nm (OD450) was measured using a spectrophotometer (BioTek, Winooski, VT).

### 3.4. Determination of Equilibrium Dissociation Constants (Kd)

As described previously (18), the binding affinity of different concentrations of H-ap25-biotin (0 nmol/L, 2 nmol/L, 5 nmol/L, 10 nmol/L, 20 nmol/L, 40 nmol/L, 80 nmol/L, 160 nmol/L) with rhHMGB1 (2 pmol/well) was estimated. The Kd was calculated using the equation Y = BmaxX/(Kd + X) with GraphPad Prism software 6.0 (GraphPad Software Inc., La Jolla, CA, USA).

### 3.5. ELASA

Ninety-six-well polystyrene microplates were coated with the anti-HMGB1 antibody (Proteintech Group, cat. 10829-1-AP, diluted 1: 250) in coating buffer (50 mM carbonate buffer, pH 9.6), followed by blocking with 3% BSA/PBST (w/v). rhHMGB1, in a concentration gradient (0, 4 nmol/L, 8 nmol/L, 16 nmol/L, 32 nmol/L, 64 nmol/L, 128 nmol/L) diluted with PBST, was added to the microplates, and the plates were incubated at 37℃ for 2 hours. After four washes with PBST, H-ap25-biotin (120 nmol/L) dissolved in SHMCK buffer was added. Following a one-hour incubation at 37℃ and four washes with PBST, streptavidin-HRP was added, and the plates were processed as described in the Aptamer Binding Assay section.

### 3.6. Cell Culture Experiments

Human acute monocytic leukemia THP-1 cells were maintained in RPMI 1640 supplemented with 10% fetal bovine serum. For the experiment, the cells were inoculated into 6-well plates at 5 x 10^5^ cells/well and cultured for 24 hours. Then, the cells were assigned to six groups: Blank; library ssDNA (4 nmol/L); H-ap25 (4 nmol/L); rhHMGB1 (final concentration of 0.8 nmol/L); rhHMGB1 (0.8 nmol/L) + library ssDNA (4 nmol/L); and rhHMGB1 (0.8 nmol/L) + H-ap25 (4 nmol/L), and treated accordingly. Polymyxin B (70 U/mL, Mpbio, California, USA) was added to neutralize residual contaminating endotoxin. Four hours later, the cells were harvested for immunofluorescence and RNA and protein extraction. The medium was collected for ELISA determination of TNF-α (R & D Systems, Minneapolis, MN, USA, cat. DY210-05), IL-1β (Invitrogen, Camarillo, CA, USA, cat. BMS224-2), and IL-6 (Invitrogen, Camarillo, CA, USA, cat. EH2IL6) concentrations.

### 3.7. Dot-Blot, South-Western Blot and Western Blot

To confirm the affinity and specificity of H-ap25 for the HMGB1 B box, rhHMGB1, rhHMGB1 A box, rhHMGB1 B box, and BSA (4 pmol in 5 μL) were dotted onto a PVDF membrane. For South-Western blot analysis, 100 µg of THP-1 cell protein (lysed in radioimmunoprecipitation assay buffer supplemented with protease inhibitor) or 4 pmol rhHMGB1 was resolved by 12% SDS-PAGE and transferred onto a PVDF membrane. After blocking in 10% (w/v) skim milk in PBST for 1 hour at room temperature, the membranes were incubated with H-ap25-biotin (40 nmol/L) or library ssDNA-biotin (40 nmol/L) at 4℃ overnight. After four washes with PBST, the membranes were incubated with streptavidin-HRP (1: 5000 dilution) for 1 hour at room temperature. The blots were visualized with the enhanced chemiluminescence detection system (ECL-Plus, GE Healthcare, Life Science, Uppsala, Sweden) after washes with PBST.

For Western blot analysis, 4 pmol rhHMGB1 or 100 µg of THP-1 cell protein was resolved by SDS-PAGE and transferred onto a PVDF membrane. Anti-HMGB1 antibody (1: 500 dilution) and anti-TLR9 antibody (Invitrogen, cat. PA5-20203, 1:500 dilution) were used as the primary antibodies. The gray scale quantification of TLR9 bands was determined using Bandscan 5.0.

### 3.8. Aptamer-Based Histochemical Staining

Mouse fibrotic lung tissue (bleomycin-induced) and fibrotic liver tissue (CCl4-induced) from previous studies were used to test aptamer-based histochemical staining, as described previously (28), except that the H-ap25-biotin concentration was 200 nmol/L. Immunohistochemistry with the anti-HMGB1 antibody (1: 500 dilution) was carried out as a positive control.

### 3.9. Immunofluorescence

To detect the activation of NF-κB, the subcellular distribution of RelA (p65) was examined immunofluorescently in THP-1 cells. The cells were attached to coverslips and fixed with methanol. Anti-RelA (p65) antibody (Proteintech Group, cat. 10745-1-AP) was used as the primary antibody, and FITC-labeled goat anti-rabbit antibody served as the secondary antibody. The nucleus was stained with propidium iodide (PI).

### 3.10. qRT-PCR

Total RNA was extracted using TRIzol reagent (Thermo, Life Technologies, Carlsbad, CA). Reverse transcription was performed with a Transcriptor First Strand cDNA Synthesis Kit (Roche Diagnostics, Indianapolis, IN, USA). The relative abundance of TNF-α, IL-1β, and IL-6 mRNA in the sample was determined using the TransStart^®^ Tip Green qPCR SuperMix (Transgen Biotech, Beijing, China) on an ABI StepOnePlus™ Real-Time PCR Detection System (Applied Biosystems, Thermo Fisher Scientific, USA). Cycle threshold values were obtained from StepOne™ Software v2.2.2 (Applied Biosystems ABI). The levels of relative TNF-α, IL-1β, IL-6, and TLR9 mRNA were calculated using the 2−ΔΔCt method, with β-actin serving as an internal control (primers are listed in Table 3). 

**Table 3.** Primers Used in qRT-PCR

Names	Sequences
**TNF-α**	5′-CGAGTGACAAGCCTGTAGCC-3′
	5′-CCAGCTGGTTATCTCTCAGC-3′
**IL-1β**	5′-TGTACCTGTCCTGCGTGTTG-3′
	5′-GAAGACGGGCATGTTTTCTG-3′
**IL-6**	5′-AGAGCTGTGCAGATGAGTAC-3′
	5′-GTCATGTCCTGCAGCCACTG-3′
**TLR9**	5′-CCTGTAGCTGCTGTCCAGTC-3′
	5′-GCACAGACTTCAGGAACAGC-3′
**β-actin**	5′- GAAGTGTGACGTGGACATCC-3′
	5′- GATCCACACGGAGTACTTG-3′

### 3.11. Statistical Analysis

Statistical analyses were performed using GraphPad Prism (v.6.01, GraphPad Software Inc., La Jolla, CA, USA). All the results are presented as the mean ± standard deviation (SD). Multiple comparisons were performed using a one-way ANOVA test followed by the Tukey-Kramer test for post hoc analysis. Differences between two groups were examined using an unpaired Student's *t-*test. P < 0.05 was considered statistically significant.

## 4. Results

### 4.1. Expression and Purification of Recombinant Human HMGB1(rhHMGB1), rhHMGB1 A Box, and rhHMGB1 B Box

**Figure 1.**
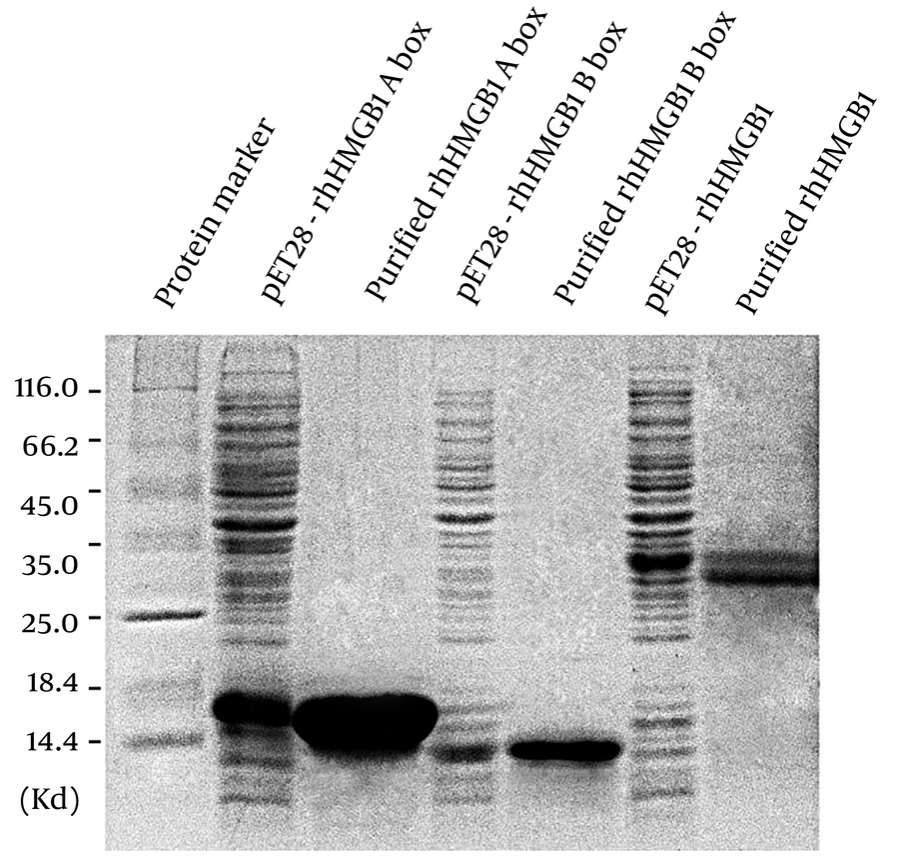
Expression and purified of rhHMGB1, rhHMGB1 A box and rhHMGB1 B box. Proteins were resolved by SDS-PAGE followed by Coomassie brilliant blue stain.

Using an *E. coli* expression system and Ni-NTA chromatography purification, we obtained recombinant human HMGB1 (rhHMGB1), rhHMGB1 A box, and rhHMGB1 B box (Figure 1). Coomassie blue staining following SDS-PAGE revealed that rhHMGB1 A box and rhHMGB1 B box showed single bands with apparent molecular masses identical to their respective calculated molecular masses. In contrast, purified rhHMGB1 showed two closely neighboring bands with similar density, as reported by Li et al. (4), who verified that both bands were biologically active rhHMGB1.

### 4.2. Selection and Characterization of the DNA Aptamer Against HMGB1

**Figure 2.**
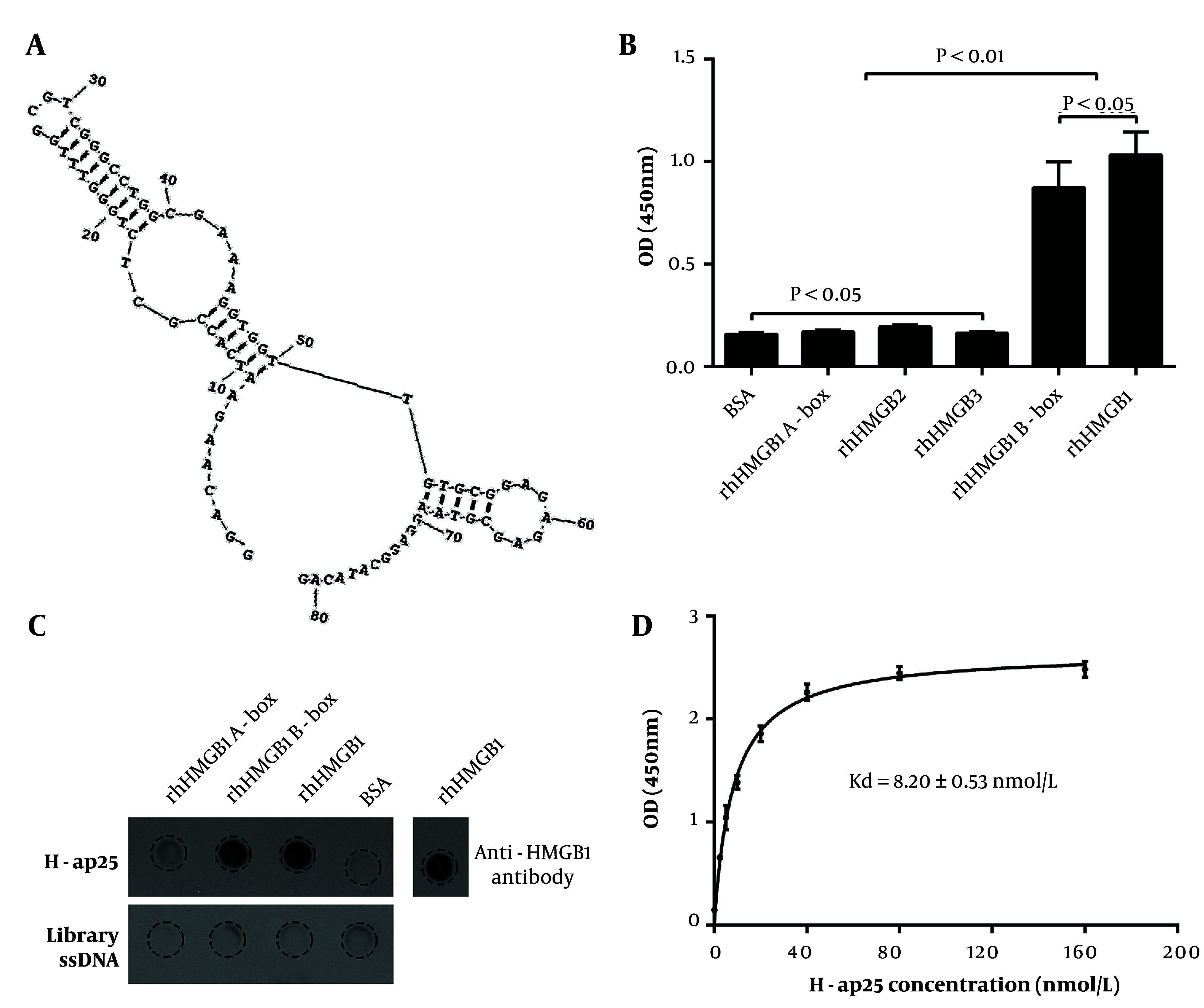
Characterization of H-ap25. The predicted secondary structure of H-ap25 presented a stable structure composed of a hairpin (9 - 50) and a stem-loops (52 - 69) linked by a T (A). H-ap25 specifically bound to rhHMGB1 and rhHMGB1 B box but not rhHMGB1 A box, as indicated by aptamer binding assay (B) and Dot-blot (C). In addition, H-ap25 does not bind to HMGB2 and HMGB3, which have high homologiesy to with HMGB1 (B). Equilibrium dissociation constant (Kd) of H-ap25 was 8.20 ± 0.53 nmol/L (D). The results are presented as the mean ± standard deviation (SD) of triplicate reactions.

Next, we performed a 15-round iterative SELEX with rhHMGB1 B box immobilized on microplates to select the DNA sequences binding to rhHMGB1 B box. Forty-eight transformed colonies were picked, and the ssDNA was prepared using asymmetric PCR with the biotin-labeled forward primer for the binding assay. Sequence analysis of the top eight aptamer candidates with higher affinity showed that they shared highly similar sequences. Secondary structure prediction showed that the individual aptamers, along with the flanking sequences, folded into similar conformations, while they failed to fold into stable conformations when the flanking sequences were excluded. We chose H-ap25, which exhibited the highest affinity, for further experiments. The sequence of H-ap25 is 5′-GGACAAGAATCACCGCTCTGGGTTTGGCGTCGGGCCTGGCG AAAGGTGGTTGTGCGGAGAGAGCGTACAGGAGGCATACAG-3′, with the predicted structure shown in Figure 2A. 

Aptamer-target binding assays with both microplate- and membrane-immobilized (dot blot) rhHMGB1, rhHMGB1 A box, and rhHMGB1 B box demonstrated that H-ap25 specifically bound to rhHMGB1 and rhHMGB1 B box, but not to rhHMGB1 A box, HMGB2, or HMGB3 (Figure 2B and C). The calculated equilibrium dissociation constant (Kd) of H-ap25 was 8.20 ± 0.53 nmol/L (Figure 2D). 

### 4.3. Application of H-ap25 in South-Western Blot, ELASA and Immunohistochemical Staining

**Figure 3.**
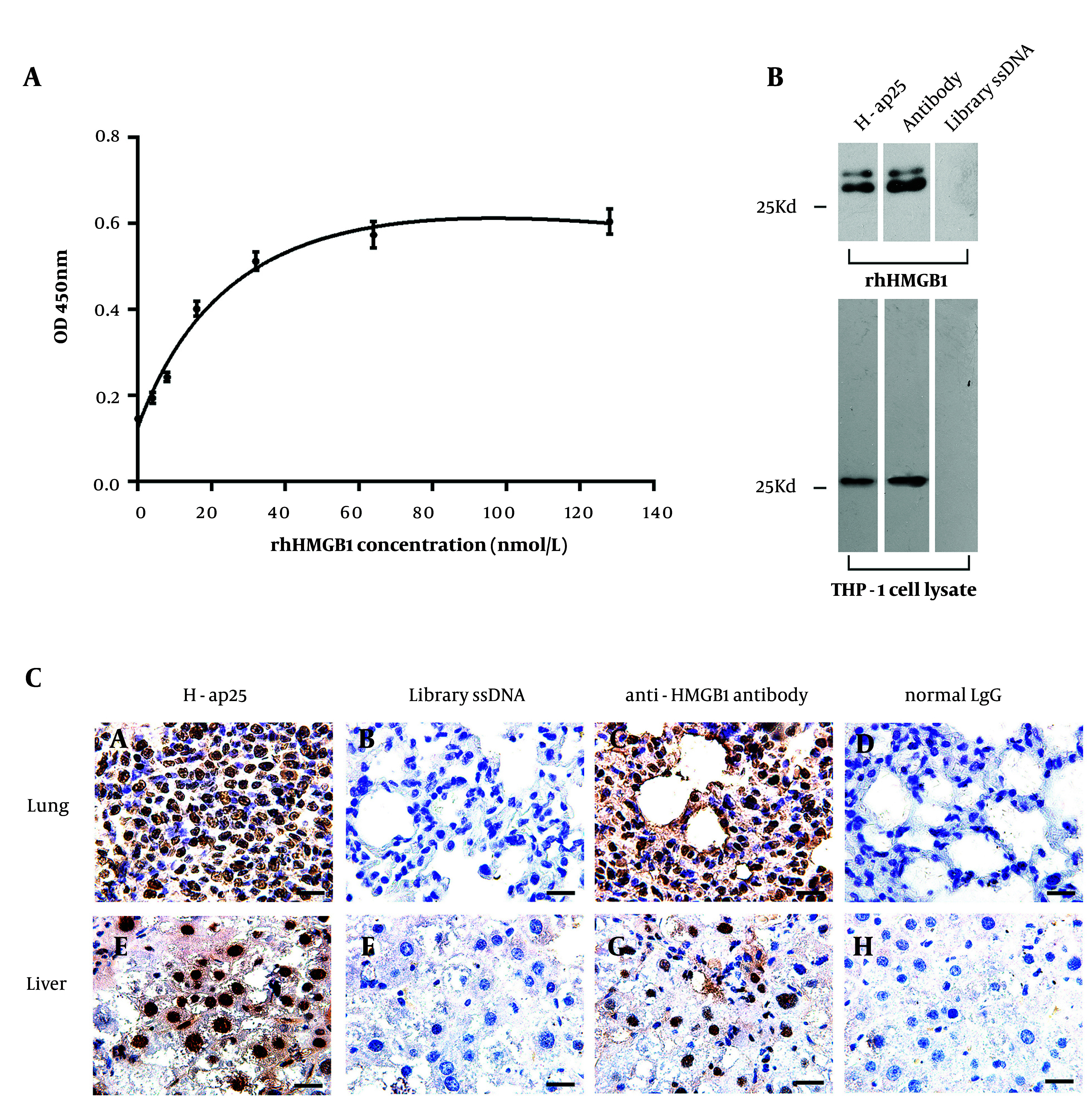
Application of H-ap25 in the detection of HMGB1. Sandwich ELASA with H-ap25- biotin exhibited a nice linear relationship between the concentration of rhHMGB1 and the optical density. The results are presented as the mean ± standard deviation (SD) of triplicate reactions (A). H-ap25-biotin specifically recognized denatured rhHMGB1 and cellular HMGB1 in South-Western blot (B). Aptamer-based histochemical staining showed specific nuclear staining both in mouse liver and lung tissues, comparable to the results of immunohistochemistry with anti-HMGB1 antibody (C). Scale bars = 20μm.

The utility of H-ap25 in detecting HMGB1 was then tested. A sandwich ELASA using an anti-HMGB1 antibody as the capturing antibody and biotin-labeled H-ap25 as the detector showed a strong linear relationship between rhHMGB1 concentrations and the optical densities (OD) when biotin-labeled H-ap25 was utilized at concentrations between 0 and 32 nmol/L (Figure 3A). In South-Western blot analysis, H-ap25 specifically recognized HMGB1 in THP-1 cell protein as well as rhHMGB1, as confirmed by Western blot with the anti-HMGB1 antibody (Figure 3B). Immunohistochemical staining with formalin-fixed, paraffin-embedded mouse lung and liver tissue was performed to test the potential applications of H-ap25. The results showed that both H-ap25 and the anti-HMGB1 antibody exhibited similar positive nuclear staining (Figure 3C). These results suggest that H-ap25 might be developed into a molecular probe for the detection of HMGB1.

### 4.4. Enhancement of H-ap25 to HMGB1 on Pro-Inflammatory Through Activing TLR9/NF-κB Pathway

**Figure 4.**
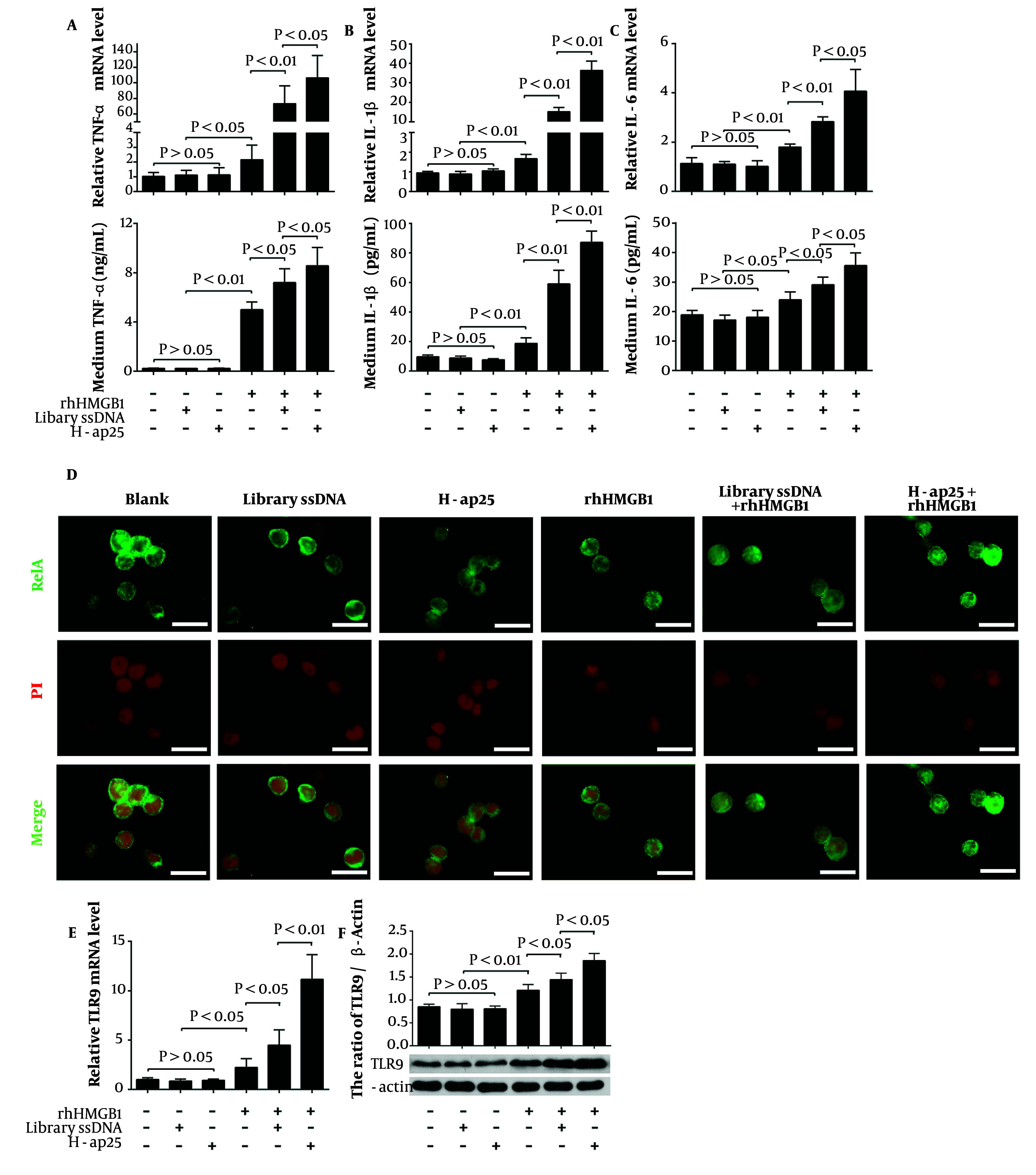
H-ap25 enhanced the pro-inflammatory response of HMGB1 in THP-1 cells. Both qRT-PCR (upper panel) and ELISA (lower panel) showed that rhHMGB1 up-regulated TNF-α (A), IL- 1β (B) and IL-6 (C) expression in THP-1 cells. These effects were markedly enhanced by both H-ap25 and library ssDNA whereas H-ap25 exhibited a stronger action than library ssDNA. Immunofluorescence demonstrated that the combination of Hap25 and rhHMGB1 exhibited a stronger effect of inducing p65 nuclear translocation than either rhHMGB1 or library ssDNA + rhHMGB1 (D). Scale bars = 40 μm. qRT-PCR (E) and Western blot (F) showed that the combination of H-ap25 and rhHMGB1 was most effective on up-regulation of TLR9 in THP-1 cell than either rhHMGB1 alone or rhHMGB1 + library ssDNA. The results of qRT-PCR and Western blot are presented as the mean ± standard deviation (SD) of four separate experiments.

Finally, we tested the influence of H-ap25 on the pro-inflammatory effects of HMGB1. THP-1 cells were used as the effector since they can express pro-inflammatory cytokines under HMGB1 stimulation. rhHMGB1 at a final concentration of 0.8 nmol/L significantly upregulated the expression of TNF-α, IL-1β, and IL-6 at both mRNA and protein levels in THP-1 cells. The combination of rhHMGB1 with either H-ap25 or library ssDNA exhibited stronger effects than rhHMGB1 alone, with rhHMGB1 + H-ap25 resulting in significantly higher expression levels of the pro-inflammatory cytokines in THP-1 cells compared to rhHMGB1 + library ssDNA (Figure 4A - C).

RelA (p65), a protein involved in NF-κB heterodimer formation, was detected by immunofluorescence to further verify the enhancing effect of H-ap25 on HMGB1. In line with the expression profile of the pro-inflammatory cytokines, HMGB1-induced RelA nuclear translocation in THP-1 cells was enhanced by both library ssDNA and H-ap25, with H-ap25 showing a stronger effect than library ssDNA (Figure 4D). 

Additionally, the possible effect of HMGB1 and/or H-ap25 on the expression of HMGB1 receptors such as the receptor for advanced glycation end products (RAGE), TLR2, TLR4, and TLR9 was determined using qRT-PCR and Western blot. The results showed that both library ssDNA and H-ap25 enhanced the upregulation effect of rhHMGB1 on TLR9 expression in THP-1 cells, with H-ap25 exhibiting a stronger effect than library ssDNA (Figure 4E and F). rhHMGB1 did not affect the expression of RAGE, TLR2, and TLR4 in THP-1 cells (data not shown).

## 5. Discussion

HMGB1 has been recognized as a potential novel diagnostic and prognostic marker in a broad spectrum of diseases, such as sepsis, arthritis, inflammatory bowel diseases, fibrotic diseases, and diabetes (2-11). Therefore, an appropriate detection method for HMGB1, particularly in body fluids, is necessary for both clinical diagnosis and scientific research. Conventionally, antibody-based methodologies such as ELISA and electrochemiluminescence immunoassay (ECLIA) are widely and conveniently used for detecting antigens in body fluids. However, certain factors present in body fluids, including IgG, can bind to HMGB1 and interfere with its detection in ELISA assays (29). Consequently, Western blot analysis remains the most accurate method for detecting HMGB1, although a few ELISA kits are commercially available. Aptamers, characterized by their low molecular weight, rapid tissue penetration, lack of immunogenicity, and affordability, can serve as excellent tools for assessing HMGB1.

In the present study, negative selection was performed with recombinant HMGB1 A box to exclude binding to possible contaminant proteins from the expression system as well as HMGB1 A box. Moreover, since HMGB2 and HMGB3 share 80% and 75% homology with HMGB1, respectively, negative selection was also performed with HMGB2 and HMGB3 to ensure the specificity of the aptamer. After a 15-round SELEX, H-ap25 emerged from the pool with a high affinity for HMGB1 (Kd = 8.20 ± 0.53 nmol/L). As anticipated, H-ap25 exhibited excellent specificity for HMGB1 without detectable affinity for HMGB2 or HMGB3.

The utilization of H-ap25 in detecting HMGB1 was preliminarily evaluated. ELISA using H-ap25 produced a perfect standard curve with rHMGB1 concentrations below 64 nmol/L and reliably detected HMGB1 at concentrations as low as 4 nmol/L. In South-Western blot and aptamer-based histochemical staining, H-ap25 demonstrated comparable results to anti-HMGB1 antibodies in detecting both human and mouse HMGB1. With further modifications of the aptamer sequence and the determination procedure, H-ap25-based detection systems might be developed into practical measurement methods for clinical and research applications.

We aimed to identify a DNA aptamer that not only recognizes HMGB1 but also antagonizes the bioactivity of extracellular HMGB1. This is why rhHMGB1 B box, rather than the full length of rhHMGB1, was chosen as the target in SELEX. Contrary to our expectations, the results of the neutralizing experiment demonstrated that H-ap25 promoted rather than inhibited the rhHMGB1-induced inflammatory response in THP-1 cells, indicating that H-ap25 enhances the pro-inflammatory ability of HMGB1. It has been reported that HMGB1 secreted by activated macrophages or released by necrotic cells has a strong pro-inflammatory effect, while finely purified recombinant HMGB1 has only weak bioactivity, highlighting the importance of synergistic factors beyond the redox status of HMGB1 (9, 30-32). Extracellular HMGB1 retains the ability to bind to various types of DNA, including host genomic, mitochondrial, and microbial DNA, which can reinforce HMGB1's activation of the innate immune response (33-35). We speculate that the ssDNA library and HMGB1 may have undergone nonspecific binding, potentially resulting in the enhancement of HMGB1 activity in the in vitro experiment. Moreover, CpG-rich DNA is a strong enhancer of HMGB1-mediated activation of TLR9 and RAGE (33, 34). H-ap25, like almost all reported aptamers, is rich in C and G, which confers a stable higher-order structure to aptamers. It is reasonable that H-ap25, mimicking oligodeoxynucleotides containing CpG motifs (CpG-ODN) and having a high affinity for HMGB1, has a stronger pro-inflammatory effect on HMGB1 than the ssDNA library. These results suggest that H-ap25 can also serve as an enhancer of HMGB1 in research.

In conclusion, H-ap25 demonstrates high affinity and specific recognition of HMGB1, with a Kd of 8.20 ± 0.53 nmol/L. Across various assays, including South-Western blot, ELASA, and immunohistochemical staining, H-ap25 exhibits promising utility in HMGB1 detection. Furthermore, compared to random ssDNA sequences (ssDNA library), H-ap25 displays enhanced potency in activating the TLR9/NF-κB pathway following its binding to HMGB1. These findings show the potential of H-ap25 as a versatile tool for both HMGB1 detection and modulation.

## Data Availability

The dataset presented in the study is available on request from the corresponding author during submission or after publication. The data are not publicly available due to a related patent is being applied for.
